# Unprotected Sex with Injecting Drug Users among Iranian Female Sex Workers: Unhide HIV Risk Study

**DOI:** 10.1155/2012/651070

**Published:** 2012-03-19

**Authors:** Khodabakhsh Ahmadi, Majid Rezazade, Mohammad Nafarie, Babak Moazen, Mosaieb Yarmohmmadi Vasel, Shervin Assari

**Affiliations:** ^1^Behavioral Sciences Research Center, Baqiyatallah University of Medical Sciences, Tehran 14548, Iran; ^2^AIDS Prevention and Control Committee, Welfare Organization State, Tehran, Iran; ^3^Welfare Organization State, Tehran, Iran; ^4^Department of Health Behavior, Medicine and Health Promotion Institute, Tehran, Iran; ^5^Universal Network for Health Information Dissemination and Exchange (UNHIDE), Tehran, Iran; ^6^Department of Psychology, Bu-Ali Sina University, Hamedan, Iran; ^7^Department of Health Behavior and Health Education, School of Public Health, University of Michigan, Ann Arbor, MI 48109, USA

## Abstract

*Purpose*. To assess the prevalence and associated factors of unprotected sex with injecting drug users (IDUs) among a sample of female sex workers (FSWs) in Iran. *Methods*. This cross-sectional study included 144 FSWs who were interviewed as a part of Unhide HIV Risk Study, a national behavioral survey focusing on various high-risk populations, including IDUs, FSWs, and Men who have Sex with Men (MSMs) in 2009. The survey was conducted in eight provinces in Iran using respondent-driven sampling. Participants' sociodemographic status, HIV knowledge, and HIV attitude were analyzed via logistic regression to determine the predictors of unprotected sex with IDU(s) during the past month. *Results*. Nineteen percent of FSWs reported at least one occasion of unprotected sex with IDU(s) in the month preceding the study. Higher educational level (OR = −0.653, 95%CI = −1.192 to −0.115), perceived HIV risk (OR = −1.047, 95%CI = −2.076 to −0.019), and perceived family intimacy during childhood (OR = −1.104, 95%CI = −1.957 to −0.251) were all independently associated with lower odds of having unprotected sex with IDU(s) in the month preceding the study. Age, marital status, living condition, HIV knowledge, and perceived behavioral control did not affect the odds of FSWs having sex with IDUs. *Conclusion*. Perceived HIV risk, which is a modifiable factor, seems to be a promising target for harm reduction interventions amongst Iranian female sex workers. Data presented here may aid in reducing or eliminating the role of sex workers as a bridge for HIV transmission from IDUs to the general population in Iran.

## 1. Introduction

Both its location amid a major regional narcotics transit route and the increasing drug injection rate have made Iran a country with a concentrated epidemic of HIV [1, 2]. Based on data from the Joint United Nations Program on HIV/AIDS (UNAIDS), a total of 92,000 (74,000–120,000) HIV positive people were living in Iran at the end of 2009 [3].

McFarland and colleagues have discussed the challenges associated with conducting research on HIV-related behaviors amongst at-risk groups, such as female sex workers (FSWs) and men who have sex with men (MSMs) in Middle Eastern countries. They argue that this may be, in part, related to sociocultural sensitivities and political restrictions [4]. Whatever the cause of this difficulty is, the consequences do not change. Though numerous studies in this field have been conducted, our understanding about HIV risk behaviors in this particular region is very limited, and this limited knowledge hinders our ability to intervene.

Female sex workers are considered an epidemiologic “core group” for transmission sexually transmitted diseases (STDs), including HIV. This is partly attributable to both the high number and rapid turnover of sexual partners and its most common consequences, such as genital trauma, resulting from the reduction in lubricating vaginal fluid due to the use of incompetent astringents. The higher rate of STD transmission in this group is also attributable to several structural and environmental obstacles that prevent FSWs from receiving preventive interventions [5, 6]. Although available data are generally limited in this area, especially in some developing countries [7, 8], high prevalence of STDs among FSWs has been reported [9].

One particular cause of STD transmission among FSWs is unprotected sexual relationships with injecting drug users (IDUs) [10], who serve as reservoirs for HIV, hepatitis C or B [11], in addition to their high-risk sexual [10] and injecting [12] behaviors. The considerable prevalence of HIV and HCV—estimated to be about 14% and 80%, respectively—among Iranian IDUs at the end of 2009 [3] suggests the large role this population has in transmission of these infections to the general Iranian population.

Although anecdotal accounts often emphasize the role of IDUs and FSWs in HIV transmission, few empirical data exist on the predictors of unprotected sex between FSWs and IDUs—two major at-risk groups for HIV infection. The aim of this study was to determine the rate and associated factors of unprotected intercourse with IDU(s) among Iranian FSWs.

## 2. Methods

This cross-sectional study included 144 FSWs who were interviewed as a part of Unhide HIV-Risk Study, a national behavioral survey of different high-risk populations, including IDUs, FSWs, and Men who have Sex with Men (MSMs) in 2009.

### 2.1. Design and Setting

This cross-sectional study used data from Iranian noninjecting FSWs who participated in UNHIDE HIV-Risk Study. This was a national survey conducted in eight different provinces of Iran: Tehran, Fars, Isfahan, Markazi, Khuzestan, Guilan, Khorasan, and Azerbaijan. The survey of the unique population was conducted by the Behavioral Sciences Research Center at Baqiyatallah University of Medical Sciences during 2009.

The study was named Unhide HIV Risk, because it was aided by an international network, Universal Network for Health Information Dissemination and Exchange (UNHIDE), to explore different aspects of risk-taking among Iranian FSWs, IDUs, or MSMs. The ultimate goal of the study was to provide evidence needed for interventions and health promotion programs aimed at the population of interest. UNHIDE is an international network which tries to increase the availability of research-based knowledge in different disciplines of public health, which can be used for policy making and program planning.

### 2.2. Ethical Procedures

Informed consent was obtained from all participants after they had been verbally assured that the information would be kept confidential, especially from the correctional system; additionally, all checklists and questionnaires were anonymous. The Ethical Review Committee of the Baqiyatallah University of Medical Sciences approved the study.

### 2.3. Participants and Sampling

All participants were noninjecting FSWs recruited from streets in the above listed provinces. Participants were selected using snowball sampling over a 7-month period in 2009. Only participants who reported no injection during their lifetime were entered into this analysis.

## 3. Interview Process

Each interview lasted up to 60 minutes. No monetary incentive was offered to the participants. University-trained research assistants interviewed our participants; training for the interviewers was conducted through a series of workshops. All participants received HIV education and free condoms.

### 3.1. Measures

Our questionnaire measured sociodemographic data (age, gender, educational level, housing, and occupational situation), family data (marital status and perceived family intimacy during childhood), HIV knowledge (knowledge about safe and unsafe sex, as well as other HIV transmission routes), HIV attitude (perceived HIV risk, perceived need for HIV education, perceived behavior control, and intention), and HIV risk behaviors.

### 3.2. Main Outcome

The following single item measure was applied to determine unprotected sex with IDU(s): “During the past month, how many times did you have sex with a male drug injector who did not use a condom?” Sexual risk taking behaviors have been assessed by a single question in prior studies [13, 14].

### 3.3. Statistical Analysis

Data were processed in the Statistical Package for the Social Sciences 13 (SPSS Inc, IL, USA) for Windows. For bivariate analysis, Mann-Whitney *U* and chi-square tests were applied. The logistic regression model was used to determine associated factors of unprotected sex among participants. Whereas none of the bivariates showed a significant association with the outcome, predictors which were supported by the literature were entered into the regression analysis. Odds ratios (ORs) and 95% Confidence Intervals (CI) are reported. Significance level was set at *P* < 0.05.

## 4. Results

Of the 144 female sex workers included in this study, 51% were married, 89% had at least a primary education, 35% were homemakers, and 21.5% reported living with friends. The data indicated that 32.6% of the FSWs reported themselves being exposed to much or too much violence by their family members; 35% reported no or little intimacy between their family members; 41.7% thought that they would never acquire HIV in their lifetime; 97% were eager to know about HIV and its transmission routes; 24% believed they had no HIV knowledge; 38% expressed that radio and television were their main means of acquiring HIV knowledge; 53% had never received face-to-face HIV education (Tables 1 and 2).

Twenty-seven Iranian female sex workers (19%) reported having sex with IDU(s) during the past month. In the bivariate analysis, marital status was not associated with this outcome. Educational level (*P* = 0.513), family intimacy (*P* = 0.093), and perceived HIV risk (*P* = 0.774) did not show any significant difference between FSWs who reported sex with IDU(s) and those who did not. HIV knowledge was not significantly different between those with and those without unprotected sex with IDU(s) (*P* = 0.513). 

In the logistic regression, higher educational level (OR = −0.018, 95%CI = −1.192 to −0.115), perceived HIV risk (OR = −0.046, 95%CI = −2.076 to −0.019), and family intimacy during childhood (OR = −0.012, 95%CI = −1.957 to −0.251) were associated with lower odds of unprotected sex with IDU(s) among Iranian noninjecting female sex workers (Table 3).

## 5. Discussion

About 20% of Iranian noninjecting FSWs reported having sex with at least one IDU during past month. Our findings suggest that a lower tendency toward unprotected sex with an IDU amongst sex workers with higher educational levels increased perceived HIV risk and family intimacy among childhood. HIV knowledge, however, failed to be predictive of sex with IDUs in our study. These findings shed more light on a previously understudied outcome—unprotected sex with IDUs committed by noninjecting Iranian FSWs

Having a higher educational level (OR = −0.018) was shown to be associated with unprotected sex with IDUs among Iranian FSWs. Although most of studies have shown an association between educational level and risky behaviors [13–15], there are studies that have reported a lack of association [16]. Based on the Strain Theory, delinquencies may occur among those who get frustrated by the inability to succeed in school [17]. The Social Control Theory also suggests that this association results from the protective effect of institutions on instilling social norms and sanctioning deviance [17]. The Primary Socialization Theory suggests that weak school bonds may increase the amount of time spent with deviant peers [18]. All these theories can be used to explain an association between education level and risk-taking. In addition, it is possible that those with lower education may overreport behavior problems, while those with higher education may underreport behavior problems [19].

Higher perceived HIV risk (OR = −0.046) was another protective factor against unprotected sex with IDU(s). Most, [20, 21] but not all, [22] studies support our findings, and this inconsistency might be explained by measurement incompatibility, subpopulation and behavioral differences, and unexamined critical factors constructing perceived risk [22] across studies. Perceived risk is a construct of the Health Belief Model (HBM), which has been widely used for HIV prevention interventions. The Health Belief Model has been utilized over time by many authors to explain high-risk sexual behaviors among commercial sex workers previously. In a study conducted on 211 male street prostitutes, between the ages of 18 and 51 in the USA, perceived HIV risk was not significantly associated with their high-risk behaviors [23]. Other studies have shown that perceived susceptibility to HIV and perceived benefits of condom use may reduce HIV risk-taking [23]. Additionally, in another study, perceived susceptibility to STDs was a predictor of condom use among FSWs in Indonesia [24].

As reported by the United Nations, the Health Belief Model is one of the models that can be used as a basis for prevention of HIV via sexual behaviors [25]. Perceived HIV risk is a complex construct because female sex workers may be seen as at risk, not because of their current behavior, but because of their past behaviors or because of their partner [25].

More family intimacy during childhood may protect FSWs from unprotected sex with IDUs (OR = −0.012). Several studies have shown negative impact of childhood violence, physical abuse [26–28], and even witnessing family violence on future engagement in high-risk behaviors [29]. The protective role of family connectedness against deviances has been widely acknowledged [30]. As it has been suggested before, strengthening family ties and family involvement may have a protective effect on sexual risk taking of girls [31].

Low efficacy of programs that only promote HIV knowledge in relation to risky behaviors has been previously reported [32]. Program planners may consider focusing on perceived HIV risk to reduce instances of unprotected sex amongst Iranian female sex workers with IDU(s). Although this study only measured individual level data, proper prevention interventions should consider interpersonal, environmental, and structural context in which sexual behaviors occur, as well [33].

Some researchers have argued that the ultimate target for any program for FSWs should be their empowerment to quit prostitution [6]. Sex work is different in different settings [34], and such diversities should be considered when a program is designed or implemented [25].

Because of its cross-sectional design, our study is not conclusive about causal relations. The considerable amount of missing data was another limitation in this study. As the data were collected via self-report, overreporting and/or underreporting are possible consequences [35]. Small sample size and the use of respondent-driven sampling may also have limited our study. In addition, we did not collect data regarding whether the IDU is a regular or temporary client of the sex worker. This study also has not measured the context in which sex has taken place. Finally, a portion of FSWs who did not reported sex with IDUs may likely be unaware of their sexual partner's injection behavior. However, due to cultural, political, and religious circumstances, few studies have been done on Iranian FSWs [36]. Therefore, even with the aforementioned limitations, this study sheds light on risk-taking behavior of FSWs, and it may help harm reduction practice in Iran. 

In conclusion, public health officials should not assume that providing educational information about HIV transmission would lead to behavioral change among sex workers. We instead suggest theory-based interventions, with perception of risk as the integral component to reducing risky behaviors and increasing condom use among Iranian female sex works.

## Figures and Tables

**Table 1:** Sociodemographic data among Iranian female sex workers who have sex with injecting drug users (*n* = 144).

Data	Number	Percent
Marital status		
Single	69	47.9
Married	74	51.4
Missing	1	0.7
Educational level		
Uneducated	13	9.0
Primary school	19	13.2
Some secondary school	67	46.3
High school diploma	28	19.4
Associated degree	9	6.3
Bachelor's degree and higher	5	3.5
Missing	3	2.1
Housing		
Personal	17	11.8
Rental	77	54.2
Father's house	37	25.7
Friend's house	6	4.2
Relatives house	5	3.5
Missing	2	1.4
Occupational status		
Student	9	6.3
Self-employee	11	7.6
Employee	11	7.6
Homemaker	51	35.4
Home service	8	5.6
Work at private companies	19	13.2
Unemployed	24	16.7
Missing	10	6.9
Living status		
Alone	11	7.6
With family members	115	59
With others	17	11.9
Missing	1	0.7

**Table 2:** Childhood trauma and HIV attitude among Iranian female sex workers who have sex with injecting drug users (*n* = 144).

Childhood trauma	Number	Percent
Exposure to violence by family members		
Never	25	17.4
Little	31	21.5
Sometimes	37	25.7
Much	34	23.6
Too much	13	9
Missing	4	2.8
Family intimacy		
Not at all	14	9.7
A little	35	24.3
Some	52	36.1
High	29	20.1
Very high	11	7.6
Missing	3	2.1
*HIV attitude*		
Perceived HIV risk		
Not at all	60	41.7
A little	38	26.4
Some	35	24.3
High	7	4.9
Very high	3	2.1
Missing	1	0.7
Perceived need for HIV knowledge		
Not at all	4	2.8
A little	11	7.6
Some	33	22.9
High	45	31.3
Very high	51	35.4
Perceived HIV knowledge		
Not at all	34	23.6
A little	31	21.5
Some	39	27.1
High	33	22.9
Very high	5	3.5
Missing	2	1.4
Source of HIV Information		
Radio and television	29	20.1
Newspaper	7	4.9
Books	5	3.5
Friends	21	14.6
Educational programs/interventions	14	9.7
Some mixing ways	46	31.9
Missing	22	15.3
Participation in HIV education programs		
Never	77	53.5
Once	15	10.4
Twice	11	7.6
More	37	25.7
Missing	4	2.8

**Table 3:** Predictors of unprotected sex with injecting drug users among Iranian female sex workers (*n* = 144).

Characteristic	OR	95%CI for (OR)	
		Lower	Upper
Higher educational level	−0.653	−1.192	−0.115
Higher family intimacy during childhood	−1.104	−1.957	−0.251
Perceived HIV risk	−1.047	−2.076	−0.019
